# Baseline neutrophil–lymphocyte ratio is associated with baseline and subsequent presence of brain metastases in advanced non-small-cell lung cancer

**DOI:** 10.1038/srep38585

**Published:** 2016-12-07

**Authors:** Young Wha Koh, Jin-Hyuk Choi, Mi Sun Ahn, Yong Won Choi, Hyun Woo Lee

**Affiliations:** 1 Department of Pathology, Ajou University School of Medicine, Suwon, Korea; 2 Department of Hematology-Oncology, Ajou University School of Medicine, Suwon, Korea

## Abstract

We examined the predictive value of neutrophil–lymphocyte ratio (NLR) by examining their association with the baseline presence and subsequent development of brain metastases in patients with stage IV non-small cell lung cancer (NSCLC). We examined the predictive value of NLR for brain metastasis in 260 stage IV NSCLC. Logistic regression models and competing risk analysis were used to determine the association of NLR with baseline and subsequent presence of brain metastases. Multivariate analysis reveals that patients with high NLR (≥4.95) had significantly more brain metastases at diagnosis than those with low NLR (Odds Ratio = 2.59, *P* = 0.01). In patients who had no baseline brain metastasis, competing risks analysis revealed that patients with high NLR showed higher cumulative incidence of subsequent brain metastases, compared to those with low NLR (*P* = 0.017). A high NLR was associated with the baseline presence or the subsequent development of brain metastases, particularly in the group with adenocarcinoma (*P* = 0.013 and *P* = 0.044, respectively). Furthermore, an increase in NLR during treatment was associated with subsequent brain metastases (*P* = 0.004). The NLR is an independent predictive factor for the baseline presence of brain metastases and subsequent brain metastases in stage IV NSCLC.

Non-small-cell lung cancer (NSCLC) with brain metastasis is the most common central nervous system malignancy, accounting for as much as 20% of all brain metastasis cases^1^. Because of advances in cancer therapy, some cancer patients can prolong their lives, resulting in a higher probability of brain metastasis. NSCLC with brain metastasis often has a dismal prognosis with median survival ranging between 4 and 6 months^2^. There are currently no validated biomarkers for patients with NSCLC to predict the development of brain metastases.

Previous studies demonstrated associations between epidermal growth factor receptor (EGFR) mutation status and the development of brain metastases^3^,^4^,^5^, however other investigator reported negative result^6^. EGFR mutated adenocarcinoma comprises up to 20–50% of adenocarcinomas^7^. Furthermore, most patients with squamous cell carcinoma have no EGFR mutation^8^,^9^. Therefore, new biomarkers for detecting brain metastasis are needed for many EGFR wild type patients. Prophylactic cranial irradiation or chemotherapy was considered in patients with advanced NSCLC^10^,^11^,^12^. If we can find new biomarkers for early identification of patients at the greatest risk of brain metastasis, we can decrease mortality among NSCLC patients by more intensive brain imaging surveillance and prophylactic treatment strategies.

Neutrophil-to-lymphocyte ratio (NLR) is a useful biomarker for systemic inflammatory disease^13^,^14^. Large-scale meta-analysis revealed that NLR was associated with an adverse clinical outcome in many solid tumors^15^. Recent studies have found that the NLR at diagnosis is a prognostic indicator in malignant tumors including NSCLC^16^,^17^,^18^,^19^,^20^,^21^. It has been proposed that the imbalance between neutrophils and lymphocytes is secondary to tumor hypoxia or necrosis and is associated with tumor growth and metastasis^22^,^23^,^24^. Systemic elimination of neutrophils reduced pulmonary and lymph node metastases in a mouse model of breast cancer^25^.

Although the biologic behavior of NLR in NSCLC has been heavily researched^18^,^19^,^26^,^27^,^28^, there are no studies exploring the association of NLR and the development of brain metastases. This retrospective study evaluated the predictive value of NLR by examining its association with the baseline presence and subsequent development of brain metastases in patients with stage IV NSCLC.

## Materials and Methods

### Patient selection

This research was approved by the Institutional Review Board of Ajou University Hospital. Inform consent was granted a waiver due to the retrospective nature of this study. All treatments were performed in accordance with relevant guidelines and regulations. We carried out a retrospective study of 260 consecutive patients who received chemotherapy for NSCLC at the Ajou University Hospital (Suwon, Korea) between 2002 and 2011. All patients in this analysis had pathologically or radiologically confirmed stage IV NSCLC. Patients using corticosteroids at baseline was excluded. Brain computed tomography (CT) or magnetic resonance imaging (MRI) scans were used to detect brain metastases. Clinical information, including age, gender, clinical TNM stage, histologic subtype, chemotherapic regimen, smoking history and the Eastern Cooperative Oncology Group (ECOG) status were evaluated for association with the baseline presence and subsequent development of brain metastases. Anemia was defined as hemoglobin <130 g/l in men and <120 g/l in women based on the World Health Organization classification.

### Neutrophil-to-lymphocyte ratio

Complete blood counts (CBC), which included total white blood cells, neutrophils and lymphocytes were obtained at the time of admission.

To evaluate the predictive value of change in NLR during treatment, we analyze a correlation of post-treatment NLR and subsequent development of brain metastases in patients with low NLR and no baseline brain metastasis. Patients with stage IV NSCLC have a terrible prognosis^2^. Potential patients for subsequent development of brain metastases should have sufficient observation period because quick deaths without brain metastasis precluded the observation of brain metastasis. Therefore, we excluded patients died before 12 months after treatment to reduce competing effect. Post-treatment NLR was obtained at subsequent development of brain metastases in patients with subsequent development of brain metastases. In patients without subsequent development of brain metastases, post-treatment NLR was obtained at 15 months after treatment because median month was 15 months for subsequent development of brain metastases. If patients died between 12 and 15 months after treatment, the NLR at last follow up was obtained. The NLR was calculated using the standard formula: NLR = ANC/ALC.

### Statistical Analysis

Receiver operating characteristic (ROC) curve analysis was used to determine the optimal cut-off value of NLR, corresponding to maximum joint sensitivity and specificity.

Our statistical analyses consisted of three steps. In the first step, to evaluate whether NLR and clinical variables have prognostic significance for brain metastasis, we first examined their association with the presence of brain metastasis at diagnosis. Associations between factors and the presence of brain metastasis were determined using logistic regression. If the test has a p-value of 0.05 or less in univariate analysis, or if it was a clinically important variable, a multivariable logistic regression analysis was performed to adjust for potential covariate imbalances.

In the second step of analyses, competing risks analyses were conducted, where death and subsequent development of brain metastases in patients who did not have baseline brain metastasis were modeled as competing events. The numerous deaths without brain metastasis in stage IV NSCLC precluded the observation of brain metastasis. The time interval to subsequent development of brain metastases in patients who did not have baseline brain metastasis was calculated from the day of diagnosis until the day of subsequent development of brain metastases.

In the third step of analyses, we analyze a correlation of post-treatment NLR and subsequent development of brain metastases in patients with low NLR and no baseline brain metastasis.

Statistical significance of differences in survival rates was assessed using the Gray test^29^. The data were analyzed using the IBM SPSS/PC version 18.0 for Windows and R version 3.1.1 (http://www.R-project.org). All *P* values are two-sided associations and *P* < 0.05 was considered statistically significant.

## Results

### Patient characteristics

The clinical characteristics of the 260 patients included in the study are summarized in Table 1. Among 260 NSCLC patients, 94 (36%) developed brain metastases, detected either at diagnosis (n = 60, 23%) or subsequently during the course of treatment (n = 34, 13%). Patient ages ranged from 30 to 84 years (median: 59.5 years), 166 were men and 94 were women. By histologic subtyping, 194 had adenocarcinoma and 66 had non-adenocarcinoma. Two hundred and five patients received platinum-based chemotherapy as first-line treatment and 55 patients received non-platinum-based chemotherapy The median follow-up time was 11.5 months (range: 1–95 months). Median NLR was 3.29 (range: 0.67–95).

### Association of NLR with brain metastasis at diagnosis

**Figure 1:**
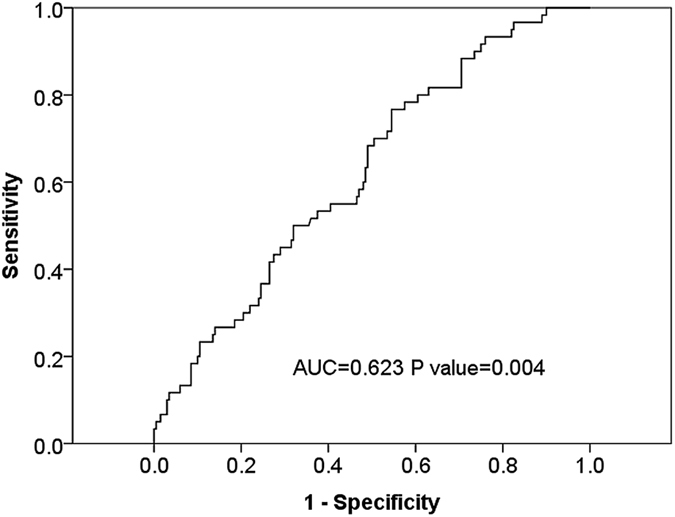
Receiver-operating-characteristic (ROC) and area under the curve (AUC) for the NLR.

ROC curves for NLR according to brain metastasis at diagnosis were generated to determine the appropriate cut-off values. The area under the curve was recorded as 0.623 (95% confidence interval [CI]: 0.545–0.700) for NLR, with an NLR value of 4.95, corresponding to the maximum joint sensitivity and specificity on the ROC curve (50% sensitivity and 68% specificity, Fig. 1).

We performed univariate and multivariate analyses in order to assess the associations between NLR and brain metastasis at diagnosis. In univariate analyses, compared to patients with low NLR, patients with high NLR (≥4.95) had significantly more brain metastases at diagnosis (31.7% vs 19.1%; Odds Ratio [OR 1.96], 95% CI: 1.08–3.57, *P* = 0.026) (Table 2). Although other risk factors were not associated with brain metastases at diagnosis in univariate analyses, we performed multivariate analyses because most risk factors were clinically important. A multivariate analysis revealed that NLR (OR: 2.59, 95% CI: 1.25–5.38, *P* = 0.01) was significant independent predictor of brain metastasis at diagnosis (Table 3). When we performed univariate and multivariate analysis, using NLR as continuous variable, NLR as continuous value was also significant predictor of brain metastasis at diagnosis in univariate and multivariate analysis (*P* = 0.021 and *P* = 0.014, respectively).

To further assess the additional prognostic information regarding NLR, we performed subgroup analyses according to histologic subtype. In adenocarcinoma, patients with high NLR had significantly more brain metastases at diagnosis (OR: 3.03, 95% CI: 1.25–7.25, *P* = 0.013). However, in non-adenocarcinoma, NLR was not associated with brain metastases at diagnosis (*P* = 0.537).

### Association of NLR with subsequent development of brain metastases in patients who did not have baseline brain metastasis

We then performed competing risks analyses to evaluate the association between NLR and subsequent development of brain metastases in patients. Therefore, cases with brain metastases at diagnosis were excluded in this analysis. In 200 patients without brain metastasis at diagnosis, subsequent brain metastasis was identified in 34 (17%) patients.

**Figure 2:**
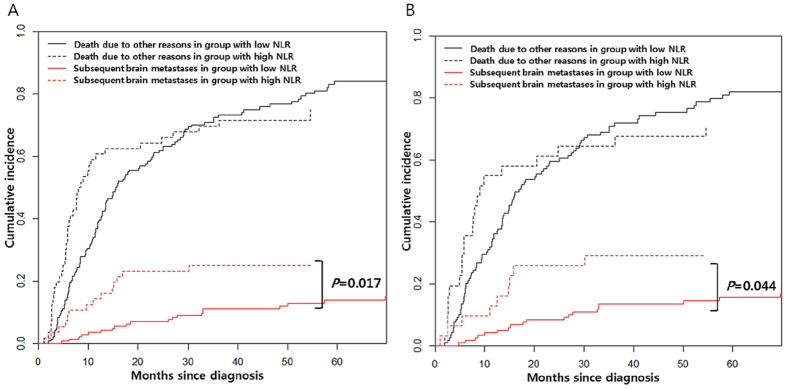
Association of NLR with cumulative incidence of subsequent brain metastasis in patients who did not experience brain metastasis at diagnosis for all NSCLC patients (**A**) and adenocarcinoma patients only (**B**).

Patients with high NLR showed higher cumulative incidence of subsequent brain metastases, compared to those with low NLR (*P* = 0.017, Fig. 2A). In the group with adenocarcinoma, patients with high NLR also showed higher cumulative incidence of subsequent brain metastases, compared to those with low NLR (*P* = 0.044, Fig. 2B).

Next, we examined a correlation of post-treatment NLR and subsequent development of brain metastases in patients with low NLR and no baseline brain metastasis. The incidence of subsequent brain metastases was significantly higher for patients with high post-treatment NLR (≥4.95) than patients with low post-treatment NLR (<4.95)(40.6% vs 12.5%, *P* = 0.004) (Table 4).

## Discussion

In the current study, we evaluated risk factors for predicting the baseline presence or the subsequent development of brain metastases in patients with stage IV NSCLC. A high NLR (≥4.95) was an independent predictor for the baseline presence of brain metastases. Competing risks analyses revealed that high NLR was associated with higher cumulative incidence of subsequent brain metastases, compared to those with patients with low NLR. Furthermore, a high NLR was associated with the baseline presence or the subsequent development of brain metastases, particularly in the group with adenocarcinoma. An increase in NLR during treatment was associated with subsequent brain metastases in patients who did not have baseline brain metastasis.

To our knowledge, we are the first to identify and validate the predictive value of NLR in NSCLC brain metastases using a competing risk model. The NLR is a readily available biomarker, which is easy to obtain from a CBC at diagnosis and will probably be one of the most inexpensive tests that can be used as a predictive model in cancer.

Although it remains unclear why a high NLR is associated with poor prognosis, it may be associated with increased neutrophil-dependent inflammation and reduced lymphocyte mediated tumor response^30^. A high NLR is detected when the absolute neutrophil count is high and the absolute lymphocyte count is low. Neutrophils can favor cancer progression or metastasis and impede the activity of lymphocytes and other immune cells, whereas the presence of tumour infiltrating lymphocytes was associated with a survival benefit^31^. Coffelt *et al*. recently reported that the depletion of neutrophils in a mouse model of breast cancer leads to a dramatic reduction in spontaneous lung metastases^25^. IL-17-producing γδ T cells induce neutrophil activation, which has the ability to suppress CD8+ cytotoxic T cells and directly promote metastatic spread.

Our results found that subsequent brain metastases happen more frequently in high post-treatment NLR (≥4.95) than in patients with low post-treatment NLR (<4.95). These results suggest that increase NLR was correlated with subsequent brain metastasis. Some previous studies reported the prognostic role of NLR changes. Early decline of NLR is associated with favorable outcomes in patients with metastatic renal cell carcinoma^32^. The posttreatment NLR change was a significant prognostic indicator for recurrence in patients with clear cell renal cell carcinoma^33^. The postoperative NLR change was an independent prognostic factor for hepatocellular carcinoma patient undergoing radiofrequency ablation^34^. Therefore, changes in NLR may help to predict a brain metastasis more accurately.

The brain is a common site of metastases for NSCLC, with 40–50% of patients developing brain metastases over the course of the disease^35^,^36^,^37^. Brain metastases are related with a generally poor prognosis, low quality of life and high economic burden, which varies depending on the treatment option^38^. Although whole-brain radiation therapy is the standard treatment for brain metastases, stereotactic radiosurgery is widely used in limited brain disease^38^. Prophylactic cranial irradiation showed a survival benefit and decreased incidence of brain metastasis in small cell lung cancer cases^39^, however there was no survival benefit in NSCLC^12^. Temozolomide has better blood-brain barrier permeability than other systemic chemotherapic agent and has modest activity in intracranial NSCLC^40^. Surprisingly, Temozolomide may have the potential to prevent the development of brain metastasis in NSCLC^10^,^11^. The NLR may help select NSCLC patients with high risk of developing brain metastases. More intensive brain imaging surveillance and prophylactic treatment strategies in this group can improve their survival and quality of life.

Our study has several limitations. First, this study had no data on EGFR mutation. Our dataset was collected between 2002 and 2011. The EGFR tyrosine kinase inhibitor was not widely used as first-line therapy during this period in Korea, therefore there were no EGFR mutational data. Some investigators found associations between EGFR mutation status and the development of brain metastases^3^,^4^,^5^,^41^. Further studies are needed to investigate the predictive role of NLR for brain metastases in NSCLC with or without EGFR mutation and the association between NLR and EGFR mutation. Second, there have been many previous studies evaluating the prognostic role of NLR in patients with NSCLC^18^,^19^,^42^,^43^. However, a well-established cutoff for NLR was not identified due to diverse study populations and various selection methods. Our identified cutoff for NLR needs to be validated in external populations. Third, our study was susceptible to bias in data selection and analysis due to retrospective design.

Using corticosteroids before measuring NLR at baseline may be a confounding factor. Small doses of corticosteroids, administered over a prolonged period of time, can induce extreme and persistent leukocytosis^44^. Corticosteroids also cause a granulocytosis primarily by a shift of neutrophils from the marginated pool to the circulating pool^45^. Previous studies described that NLR prognostic value can be used independently of prior use of corticosteroids in advanced prostate cancer and melanoma^46^,^47^. Cedrés *et al*. excluded patients taking chronic corticosteroids in prognostic analysis of NLR of NSCLC^48^. Our study also excluded patients taking corticosteroids to reduce the confounding effect.

In summary, our results suggest that a high NLR is an independent predictive factor for the baseline presence of brain metastases and subsequent brain metastases in patients who did not have baseline brain metastasis in NSCLC, particularly in the group with adenocarcinoma. The NLR can be used to identify a subgroup of adenocarcinoma patients who are at a high risk for brain metastasis, and who may benefit from aggressive chemotherapy or radiotherapy. More multicenter studies and larger sample sizes are needed to validate predictive value of NLR for subsequent brain metastases in NSCLC.

## Additional Information

**How to cite this article**: Koh, Y. W. *et al*. Baseline neutrophil–lymphocyte ratio is associated with baseline and subsequent presence of brain metastases in advanced non-small-cell lung cancer. *Sci. Rep.* **6**, 38585; doi: 10.1038/srep38585 (2016).

**Publisher's note:** Springer Nature remains neutral with regard to jurisdictional claims in published maps and institutional affiliations.

## Figures and Tables

**Table 1:** Demographic and clinical characteristics of patients.

Characteristic at diagnosis	No. of patients (%)
Age, median (range) (years)	59.5 (30–84)
Male gender	166 (63.8%)
cT stage	
T1–2	104 (43.5%)
T3–4	135 (56.5%)
cN stage	
N0–1	71 (29.7%)
N2–3	168 (70.3%)
Histologic subtype	
Adenocarcinoma	194 (74.6%)
Non- adenocarcinoma	66 (25.4%)
Anemia present	136 (52.3)
First-line therapy	
Platinum-based chemotherapy	205 (78.8)
Non platinum-based	55 (21.2%)
Smoking history present	166 (63.8%)
ECOG performance status, ≥2	25 (9.6%)

The Eastern Cooperative Oncology Group, ECOG.

**Table 2:** Univariate logistic regression analysis for association of biomarkers with presence of brain metastasis at baseline.

Variables	Brain metastasis negative	Brain metastasis positive	OR (95% CI)	*P* value
Age				0.683
Age < 70	162 (76.4%)	50 (23.6%)	1	
Age ≥ 70	38 (79.2%)	10 (20.8%)	0.85 (0.39–1.83)	
Gender				0.188
Male	132 (79.5%)	34 (20.5%)	1	
Female	68 (72.3%)	26 (27.7%)	1.48 (0.82–2.67)	
Histologic subtype				0.795
Adenocarcinoma	150 (77.3%)	44 (22.7%)	1	
Non- adenocarcinoma	50 (75.8%)	16 (24.2%)	1.09 (0.56–2.10)	
cT stage				0.236
T1–2	79 (76%)	25 (24%)	1	
T3–4	111 (82.2%)	24 (17.8%)	0.68 (0.36–1.28)	
cN stage				0.215
N0–1	60 (84.5%)	11 (15.5%)	1	
N2–3	130 (77.4%)	38 (22.6%)	1.59 (0.76–3.33)	
Anemia				0.319
None	92 (74.2%)	32 (25.8%)	1	
Present	108 (79.4%)	28 (20.6%)	0.74 (0.41–1.32)	
First-line therapy				0.638
Platinum-based	159 (77.6%)	46 (22.4%)	1	
Non platinum-based	41 (74.5%)	14 (25.5%)	1.18 (0.59–2.35)	
Smoking history				0.48
None	70 (74.5%)	24 (25.5%)	1	
Present	130 (78.3%)	36 (21.7%)	0.80 (0.44–1.46)	
ECOG performance status				0.54
<2	182 (77.4%)	53 (22.6%)	1	
≥2	18 (72%)	7 (28%)	1.33 (0.52–3.36)	
NLR				0.026
<4.95	144 (80.9%)	34 (19.1%)	1	
≥4.95	56 (68.3%)	26 (31.7%)	1.96 (1.08–3.57)	
NLR (continuous)			1.043 (1.006–1.082)	0.021

**Table 3:** Multivariate logistic regression analysis for association of biomarkers with presence of brain metastasis at baseline.

Variables		OR (95% CI)	*P* value
Age	<70 vs. ≥70	0.73 (0.27–1.96)	0.541
Gender	Male vs. Female	2.45 (0.89–6.37)	0.082
Histologic subtype	Adenocarcinoma vs. Non- adenocarcinoma	1.24 (0.57–2.65)	0.591
cT stage	T1–2 vs. T3–4	0.65 (0.33–1.28)	0.216
cN stage	N0–1 vs. N2–3	1.43 (0.65–3.13)	0.366
Anemia	None vs. Present	0.60 (0.30–1.21)	0.156
First-line therapy	Platinum-based vs. Non platinum-based	1.11 (0.44–2.76)	0.823
Smoking history	None vs. Present	0.57 (0.20–1.57)	0.277
ECOG performance status	<2 vs. ≥2	2.24 (0.47–10.5)	0.305
NLR	<4.95 vs. ≥4.95	2.59 (1.25–5.38)	0.01
NLR (continuous)		1.05 (1.01–1.09)	0.014

The Eastern Cooperative Oncology Group, ECOG.

**Table 4:** Correlation of post-treatment NLR and subsequent development of brain metastases in patients with low NLR and no baseline brain metastasis.

	subsequent brain metastases		P value
	Present	Absent	
Post-treatment NLR			0.004
<4.95	7 (12.5%)	49 (87.5%)	
≥4.95	13 (40.6%)	19 (59.4%)	
